# Coagulase‐Negative Staphylococci (CoNS): An Emerging Threat, Antimicrobial Resistance, and Health Concern

**DOI:** 10.1155/ijm/6678972

**Published:** 2026-10-14

**Authors:** Anushri Keshri, Shivani Choudhary, Anil Kumar, Indu Singh, Haritima Singh, Divakar Sharma, Arun Kumar, Sushma Verma, Arun Ratn

**Affiliations:** ^1^ Department of Biotechnology Holy Cross Women′s College Ambikapur India; ^2^ Department of Microbiology Graphic Era (Deemed to Be) University Dehradun India geu.ac.in; ^3^ Department of Allied and Healthcare Saraswathi Institute of Medical Science Hapur India; ^4^ Department of Microbiology Veer Chandra Singh Garhwali Government Institute of Medical Science and Research Srinagar India; ^5^ Dr. DIMY Bioresearch Solution Dehradun India; ^6^ Department of Biotechnology Graphic Era (Deemed to Be) University Dehradun India geu.ac.in; ^7^ Department of Biotechnology School of Basic and Applied Sciences Shri Guru Ram Rai University Dehradun India; ^8^ Noida Institute of Engineering and Technology (Pharmacy Institute) Greater Noida India; ^9^ Department of Zoology Sanatan Dharm College Muzaffarnagar India

**Keywords:** antimicrobial resistance (AMR), biofilm formation, coagulase-negative staphylococci, One Health, surveillance, therapeutic strategies

## Abstract

Coagulase‐negative staphylococci (CoNS) have evolved from a harmless commensal group to an alarming threat to patient, environmental, and population health due to their challenging treatment and their impact on health outcomes. The widespread presence of CoNS in these environments poses a public health concern due to various virulence factors, biofilm formation, and toxin production, which contribute to pathogenicity and antimicrobial resistance (AMR). Major clinical manifestations include device‐related infections, bloodstream infections, and neonatal sepsis. Nonclinical reservoirs, such as soil, water, animals, and food‐processing environments, are considered critical sources of resistance gene flow. The mechanisms of resistance include the *mecA* gene and a variety of *SCCmec* elements, as well as horizontal gene transfer via plasmids and transposons, and tolerance via biofilm formation. This review summarizes current findings on the epidemiology, AMR mechanisms, biofilm‐linked pathogenicity, diagnostic advances, and One Health implementations of CoNS across nonclinical and clinical contexts, while recognizing priorities for therapeutic and surveillance development. Here, the reviewed literature suggests considerable variation in drug resistant CoNS and distribution landscapes across nonclinical and clinical contexts, with MGEs, biofilm establishment, and environmental repertoires reflecting imperative determinants in the dissemination and persistence of AMR. An analysis of the existing literature indicates that genetic links between clinical and environmental isolates remain insufficiently established and that surveillance data are lacking; accordingly, future research should focus on these gaps. Addressing the threat of CoNS requires enhanced surveillance, improved diagnostic precision, and the development of novel antimicrobial and antibiofilm strategies. Future research should focus on epidemiological surveillance, innovation in therapy, and systems‐based approaches, which consider the interrelationship of human, animal, and environmental health.

## 1. Introduction

### 1.1. Emerging Significance of CoNS and Beyond Harmless Commensals

Coagulase‐negative staphylococci (CoNS) traditionally were harmless commensals, widespread on the skin and mucous membranes of the human body, and dismissed as contaminants when isolated in the clinical laboratory [1]. CoNS are a wide range of staphylococcal species that are not similar to *Staphylococcus aureus*, and their significant distinction is the inability to clot plasma [2]. CoNS are traditionally considered commensal organisms; they inhibit the colonization of pathogenic bacteria such as *S. aureus* and *P. aeruginosa* by competing for space and nutrients. A few of them produce antimicrobial compounds, like bacteriocins and other antimicrobial peptides (AMPs) that inhibit the growth of other pathogens. Their persistent presence on the skin and mucous membranes maintains microbial stability and reduces the possibility of opportunistic infections [3]. CoNS can stimulate immune cells to trigger a well‐controlled response and thus are less capable of causing chronic inflammation. Additionally, their action promotes the synthesis of host‐derived antimicrobial molecules, such as defensins, as an additional layer of defense. Some strains of *Staphylococcus epidermidis* can produce anti‐inflammatory compounds capable of guarding against inflammatory skin disease like eczema [4]. In animal hosts, CoNS contribute to gut health by increasing the diversity of microorganisms within the gastrointestinal tract. This could lead to improved digestion and reduced risks of intestinal infection. Within the oral cavity, these bacteria may serve as protective agents by competing with harmful species such as *Streptococcus mutans*, an important etiologic agent of dental caries, thereby reducing the risk of tooth decay [5].

Although they were historically classified as nonvirulent colonizers, epidemiological studies have now shown that they contribute significantly to the list of prominent opportunistic pathogens, particularly in nosocomial infections involving indwelling medical devices, bloodstream infections (BSIs), and infections in immunocompromised patients [1, 2]. *S. epidermidis*, earlier considered a harmless skin inhabitant, later became a primary cause of device‐related infections, which is due to its ability to form protective biofilms and resist antibiotics [6, 7]. In recent decades, advances in clinical and molecular microbiology have significantly changed the paradigm and shown that CoNS are emerging as multidrug‐resistant opportunistic pathogens in clinical, environmental, and ecological settings.

CoNS have a growing tendency to acquire and transmit resistance genes by horizontal gene transfer (HGT), diminishing the choice of treatment options and making infection management difficult. Genomic and molecular research indicates that CoNS exhibit high levels of genetic plasticity and adaptability, enabling rapid evolution under selective antibiotic pressure. *S. epidermidis*, *Staphylococcus saprophyticus*, and *Staphylococcus haemolyticus* are common CoNS species that have been consistently reported as the major causes of biofilm‐associated infections through medical devices, neonatal sepsis, and urinary infections [8, 9]. Despite accumulating research evidence clarifying the pathogenic nature of CoNS, the literature remains primarily focused on *S. aureus*; consequently, CoNS often receive limited attention, particularly in the context of antimicrobial resistance (AMR) and their clinical impact in drug‐resistant infections. An allied initiative to disseminate the status of CoNS epidemiology, resistance, and clinical implications is essential for diagnostic, therapeutic, and public health measures [10]. Moreover, new environments where CoNS are being recovered, such as wildlife, food‐processing plants, soil, and water systems, reinforce the role of CoNS as clinical sources and spread AMR outside clinical settings [11]. The One Health approach highlights the interdependence of animal, human, and environmental health and requires a renewed emphasis on these environmental reservoirs, where CoNS evolve, adapt, and spread beyond the clinic [12]. This narrative review consolidates advances in AMR mechanisms, biofilm biology, genomic studies, and the One Health paradigm, which has been indispensable in elucidating the implications of CoNS as alarming pathogens and AMR spread.

## 2. Taxonomic Classification and Clinical Adaptability of CoNS

CoNS are a heterogeneous group of catalase‐positive, gram‐positive cocci that thrive in both aerobic and low‐oxygen conditions. They differ from the virulent *S. aureus* because they cannot synthesize the coagulase enzyme. [13–15]. Microscopically, these cocci are nonmotile, nonspore‐forming, and typically found in clusters [16, 17]. The *Staphylococcus* genus consists of a total of 47 recognized species and 23 subspecies, of which 38 are coagulase‐negative [18, 19]. In recent years, several new CoNS species have been isolated from human clinical samples, such as *S. jettensis*, *S. massiliensis*, *S. petrasii* (including two subspecies), and *S. pettenkoferi* [20]. The morphology of CoNS is smooth, lustrous, and opaque colonies with diameters between 0.5 and 1.5 *μ*m after incubation for 48–72 h, although some strains exhibit mucoid textures due to the production of extracellular polysaccharides [21, 22]. *S. xylosus* and *S. chromogenes* produce orange or yellow pigments, whereas others are nonpigmented or exhibit pale colors, depending on nutrient availability and oxygen levels [23, 24]. Hemolysis on blood agar also differentiates species; *S. lugdunensis* and *S. haemolyticus* often exhibit distinct, clear *β*‐haemolytic zones that resemble those of more pathogenic *S. aureus* [25] (Figure 1). These genomic and phenotypic features facilitate the clinical adaptability of CoNS by allowing survival under distinct environmental scenarios, colonization of medical devices and host tissues, adaptation in response to antimicrobial exposure, and persistence inside biofilms.

**Figure 1:**
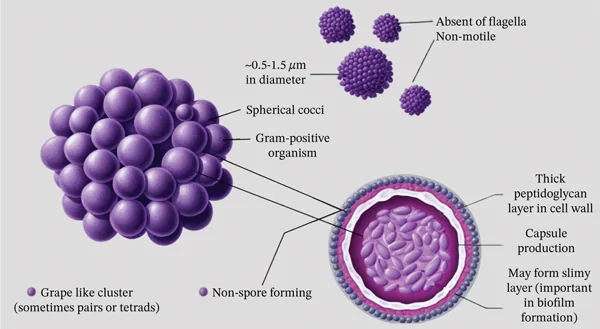
Morphological and structural features of CoNS highlighting grape‐like clusters, gram‐positive cocci with thick peptidoglycan walls, nonmotility, and capsule production associated with biofilm formation.

## 3. Epidemiology and Distribution of CoNS—Nonclinical and Clinical Settings

CoNS species are not limited to hospital settings; they are also prevalent in various nonclinical settings such as indoor air, public places, farms, food items, water bodies, soil, and surfaces [26]. Their widespread presence, along with increasing antibiotic resistance, has drawn attention and is considered a potential public health concern [27]. A recent study has identified the heavy load of CoNS in indoor air spaces, which suggests they are potent reservoirs of bacteria and revealed differences in their antibiotic resistance profile and potential health risks posed by microbial contamination [28]. CoNS are isolated from surfaces and the air of livestock and poultry farms [29]; therefore, it is of utmost importance to mitigate the threat posed by the spread of these CoNS. Furthermore, *S. epidermidis* and *S. saprophyticus* are found in freshwater and soil environments that degrade organic matter and promote biofilm formation, which directly influences the quality of aquatic ecosystems [30]. Culture‐based and metagenomic studies indicate high counts of *S. epidermidis*, *S. haemolyticus,* and *S. hominis* on skin and frequently touched surfaces of homes, schools, and trains. Studies have reported the presence of *S. sciuri, S. xylosus*, and other related species in soil, freshwater environments, livestock, and food‐processing environments [20]. Dairy foods and raw or processed foods often have particular species of CoNS involved in fermentative processes, whereas other species might serve as indicators of contamination or spoilage [31]. Additionally, CoNS are found on public and indoor surfaces, such as waiting rooms, door handles, mobile phones, and domestic articles; the presence of CoNS in these settings also poses greater challenges for infection control [32]. Research revealed higher drug resistance among staphylococcal strains from public restrooms [33]. Similar resistance patterns were found in *S. haemolyticus*, *S. aureus,* and *S. epidermidis* across different restrooms within the same facility, indicating potential transmission of resistance traits and highlighting public restrooms as reservoirs of pathogenic and nonpathogenic antibiotic‐resistant bacteria [33]. A study also highlighted the widespread presence of multidrug‐resistant CoNS in both hospital and urban belt environment settings; among them, isolates from hospitals showed a notably higher level of multidrug resistance (MDR) [34]. A notable characteristic of MDR CoNS is their ability to form robust biofilms across natural and artificial environmental surfaces [35]. CoNS extracellular polymeric substances (EPS) give strength to the biofilm matrix, offer anchorage to other microbial populations, and form sheltered protective niches for microbes against antibiotics and drying [22, 36].

### 3.1. Commensal and Beneficial Features of CoNS

CoNS are conventionally identified as commensal microbes that colonize the mucous membranes and skin, contributing to microbial homeostasis. They can impede the colonization of many pathogenic bacteria, including *P. aeruginosa and S. aureus*, via competition for nutrients and space. Few CoNS isolates also secrete bacteriocins and other AMPs that can hamper competing microbes. Their consistent presence on epithelial surfaces may thus facilitate conservation of protection and microbial stability against opportunistic pathogens [3]. CoNS may also interact with the host immune system by triggering regulated immune responses and inducing the generation of host‐driven antimicrobial molecules, including defensins. Some *S. epidermidis* strains secrete anti‐inflammatory substances that may provide defense against inflammatory skin scenarios [4]. In animal hosts, CoNS have also been linked to gut microbial diversity, whereas oral CoNS may facilitate defense against cariogenic microbes via microbial competition [5]. CoNS thus demonstrate a dual biological function: they frequently exist as relatively harmless or beneficial members of the microbiota; however, under particular environmental and host scenarios, they can transform into opportunistic pathogens. Their capability to form biofilms, colonize surfaces, acquire AMR and virulence factors, and adapt to antibiotic exposure can drive this transformation. Hence, the clinical relevance of CoNS must be considered setting‐dependent, representing the equilibrium between their commensal roles and their capability to cause sustained infection in susceptible hosts.

### 3.2. Clinical Prevalence and Infections of CoNS

This was a paradigm shift for CoNS, changing them from background flora to clinically relevant opportunistic pathogens, which played a central role in hospital‐acquired infections [37]. Researchers have looked outward, studying the occurrence and activity of CoNS in nonclinical settings where they can act as reservoirs and transmitters of AMR [2, 38]. Pathogenicity of CoNS is due to their exceptional capacity to form biofilms on inanimate surfaces, which protects them from host immune responses and antimicrobial agents, resulting in chronic infections [8]. CoNS infections reflect a major prevalence ranging from 15%–75% in hospital‐ and healthcare‐associated infections (HAIs), specifically BSIs and medical device‐linked infections [39, 40]. Their clinical burden is particularly apparent in neonatal intensive‐care settings, where CoNS have been shown to account for up to around 66% of late‐onset sepsis episodes among low‐birth‐weight and preterm infants, commonly in link to catheter use [6, 41].

### 3.3. BSIs and Catheter‐Related Bloodstream Infections (CRBIs)

BSI is the most clinically imperative characteristic feature of CoNS infection, specifically among individuals with intravascular catheters and other indwelling medical devices. *S. haemolyticus* and *S. epidermidis* are predominantly linked to medical device‐related BSIs, as their EPS and surface‐linked adhesins provide anchoring to catheter materials, followed by biofilm development [23, 42, 43]. Once settled on a medical device, CoNS biofilms facilitate amelioration from the host immunity and mitigate antimicrobial entry, resulting in treatment failure and persistent bacteremia. CRBSIs are thus imperative clinical outcomes of CoNS biofilm establishment [8, 23, 42–44]. Treatment primarily requires specific antimicrobial treatment in combination with evaluation of the infected medical device, and replacement or removal of the catheter may be essential when device‐linked biofilm is potentially suspected or when CoNS infection persists. Insufficient source control or delayed detection can lead to recurrent bacteremia, long‐term hospitalization, and elevated healthcare load [36, 45].

### 3.4. CoNS Infections in Immunocompromised Patients

Immunocompromised patients represent an imperative population at greater risk for invasive CoNS infections because the compromised immune system of the host can provide for the transformation of these microorganisms from colonization to opportunistic infection [1, 2]. Patients with hematological disorders, malignancies, hematopoietic stem‐cell or solid‐organ transplantation, and those who have undergone long‐term immunosuppressive treatments may be specifically susceptible [1, 2]. The risk is further enhanced when immunosuppression is followed by implantation of invasive medical devices including prosthetic materials, central venous catheters, urinary catheters, or other implanted devices [6, 7]. In these environments, CoNS can attach to device surfaces and develop biofilms that facilitate persistence and mitigate susceptibility to antimicrobial treatment [23, 42, 44]. On the contrary, infections may present as device‐linked infections, persistent bacteremia, or other invasive disorders, and treatment can be complicated by AMR and the difficulty of attaining potential source control [8, 44].

### 3.5. Device‐Mediated Infections

Intravenous catheters, prosthetic cardiac valves, orthopedic implants, cerebrospinal fluid shunts, and other medical devices may be infected by CoNS species, most frequently *S. epidermidis* and *S. haemolyticus*. Recent studies have shown that CoNS accounts for no more than 66% of late‐onset sepsis in preterm and low birth weight babies, which is often linked to the use of catheters [6, 41].

### 3.6. Neonatal Sepsis

Neonatal sepsis remains one of the significant health concerns worldwide, and CoNS is the key pathogen leading to late‐onset sepsis in preterm infants. They can invade the bloodstream and colonize the surfaces of the skin and mucosa of patients who have undergone prolonged catheterization and mechanical ventilation [45]. To date, epidemiological studies of various NICUs demonstrate the highest prevalence rates of CoNS as the causative agents of neonatal sepsis and high clinical and public health consequences, such as prolonged hospital stay, increased antibiotic use, and death rates [36].

### 3.7. Comparative Analysis of Clinical Versus Nonclinical Isolates

Epidemiological and microbiological studies have documented the presence of CoNS in clinical and nonclinical settings, and infections exhibit diverse patterns of AMR. Clinical CoNS isolates prevalently demonstrate substantial levels of AMR relative to isolates from animal and environmental reservoirs, which may marginally represent continued antimicrobial exposure in clinical systems [46, 47]. Nevertheless, MDR and resistant CoNS have also been observed in public and nonclinical contexts, encompassing that these repertoires may provide the dispersion and persistence of AMR [48–50]. Methicillin resistance in CoNS is primarily induced by the *mecA* gene, which is frequently carried within SCCmec MGEs and encodes the modified PBP2a [51]. A study from Nepal reported that 84.6% of clinical isolates were penicillin‐resistant, 55.8% cotrimoxazole‐resistant, and 46.2% ciprofloxacin‐resistant, with 73.1% being methicillin‐resistant [52]. In London, studies found multidrug‐resistant strains in public settings and noted their ability to serve as reservoirs for resistance genes [48, 49]. Likewise, another study on environmental pig farms detected high rates of resistance to multiple antibiotics, including last‐resort antibiotics [50]. Although hospital CoNS strains frequently exhibit greater resistance under selective antibiotic pressure, nonhospital strains still remain a contributor to the dissemination of resistance genes. Surveillance programs and effective stewardship of antibiotics are crucial for preventing resistance in hospitals and the community [37] (Table 1).

**Table 1:** Comparative analysis of clinical v/s. nonclinical isolates of CoNS.

Parameter	Clinical CoNS isolates	Nonclinical CoNS isolates (environmental, animal, food, water)	Reference
Primary sources	Blood‐borne infections, catheter or implant surfaces, immunocompromised hosts, and neonatal units;	Soils, water bodies, effluents, farm animals, pets, and food‐processing surfaces.	[33]
Antibiotic exposure pressure	Repeated use of antibiotics, invasive devices, and reservoirs in hospitals;	Low‐to‐moderate subinhibitory antibiotic antagonists in soils/water and use of veterinary antibiotics.	[53, 54]
Prevalence of methicillin resistance (*mecA/*SCC*mec*)	Very high prevalence: either isolated or other combinations of multiple SCC*mec* types (III, IV, V) are abundant, and co‐occurrence of SCCmec variants by the same strain occurs.	Less prevalent but increasing, environmental isolates still act as *mecA* depositories, and identical SCC*mec* types have been reported between environmental and clinical interfaces.	([55]; [2]; [52]; [28])
Multidrug resistance profiles	Broad resistance to *β*‐lactams, glycopeptides, aminoglycosides, macrolides, fluoroquinolones; MDR patterns frequently observed in *S. epidermidis* and *S. haemolyticus.*	Narrower resistance spectra; fewer MDR strains; however, the presence of mobile resistance determinants enables future transfer.	[50]
Biofilm formation ability	Tremendously good biofilm formation: most clinical isolates generate sturdy biofilms, a significant cause of catheter‐related bloodstream infections and treatment failures.	Capable of promoting persistent survival and genetic transfer, but with a more negligible clinical effect than on the environment (e.g., pipelines, food equipment).	([56]; [57])
Dominant species	*S. epidermidis*, *S. haemolyticus*, *S. saprophyticus* (UTIs), infrequent emergent species;	Wide species encompassing environmental *S. saprophyticus* staphylococci with changes in pathogenic potential.	[58]
Resistance gene mobility	Already a high density of horizontal gene transfer (plasmids, transposons, integrons) because of hospital settings rich in biofilms;	High ecological mixing sites in soil/water biofilms facilitate gene exchange at relatively slower rates; environmental strains tend to have early variants of ARGs.	[59]
Public health risk	Immediate and severe‐ persistent infections, neonatal sepsis, bloodstream infections, high mortality rates, treatment failure;	Indirect, but significant‐ ARG evolution and transfer into clinical strains (One Health significance). Reservoirs: Many weapons of ARG development and transmission to clinical strains exist.	[60]
Diagnostic visibility	Positive with WGS/PCR; challenging in culture because of the ambiguity between colonizers and pathogens;	Biofilms also pose challenges for ARG detection; ARGs are frequently underdetected, and metagenomics is increasingly used to identify ARG hotspots in soil/water ecosystems.	[61]
SCC*mec* diversity and evolution	Massively diverse, highly structurally varied; extensively acquired through nonclinical reservoirs via HGT	Novel or variant SCCmec elements are found in the reservoir; more and more frequently, events of environmental transfer are reported with the help of WGS.	[61]
Role in One Health AMR transmission	Location of clinical appearance of resistance.	Origin of emergent resistance genes, environmental‐animal‐human gene exchange, as shown by genome research	[62]

## 4. Resistance to Antibiotics of Nonclinical CoNS

Among infection‐related healthcare‐associated CoNS, including methicillin‐resistant and other drug‐resistant isolates, MDR is frequently reported [29, 47]. Researchers found that *S. epidermidis* was the most widespread of the clinically significant CoNS strains, followed by *S. haemolyticus* and other species. Methicillin‐resistant coagulase‐negative staphylococci (MR‐CoNS) had a reduced or heterogeneous susceptibility to vancomycin, suggesting that therapeutic challenges may persist despite vancomycin being the drug of choice [63]. Cerebrospinal fluid samples from pediatric patients with bacterial meningitis at 13 hospitals in China (2016‐2018) showed that the key pathogens were *S. epidermidis*, *E. coli*, and *S. pneumoniae*, which showed that methicillin resistance in CoNS was high, underscoring challenges in empirical treatment and the critical need of a specific antibiotic regimen [64]. Our recent research also revealed the widespread presence of multidrug‐resistant CoNS on frequently touched surfaces in hospitals and urban environments, and half of the cefoxitin‐resistant CoNS isolates carried the *mecA* gene, with SNP analysis highlighting significant genetic variation [28] (Figure 2).

**Figure 2:**
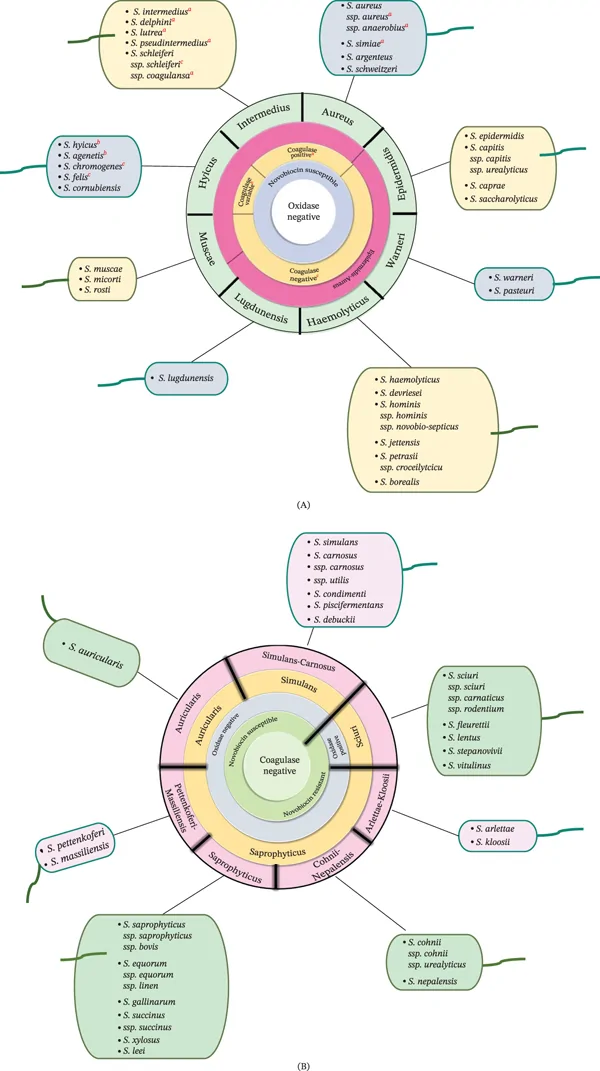
(A) Phylogenetic and biochemical classification of coagulase‐positive and coagulase‐negative *Staphylococcus* species based on oxidase reaction and novobiocin susceptibility. (B) Classification of CoNS species grouped by novobiocin resistance, oxidase activity, and phylogenetic relationships. (a: Coagulase‐positive; b: coagulase‐variable; c: coagulase‐negative).

### 4.1. Mechanisms of AMR Acquisition in CoNS

#### 4.1.1. Genetic Basis of Resistance: mecA Gene and SCCmec Elements

SCC*mec* is a notable and significant mobile genetic element associated with methicillin resistance in CoNS and MRSA. It carries the *mecA* gene, which encodes an alternative of penicillin‐binding protein (PBP2a) with low affinity for *β*‐lactams [65] and is considered a major mechanism of methicillin resistance in both clinical and environmental CoNS. They can transfer the resistance trait between different staphylococcal species, which not only enables the emergence of MRSA but also promotes the persistence of resistance in CoNS populations in nonclinical environments [17]. The presence of diverse SCC*mec* Types I to V elements in CoNS further reflects its genetic flexibility and evolutionary potential, which differ in their structural composition and resistance profiles [61]. *S. epidermidis* most recurrently harbors SCC*mec* types III and IV, often co‐occurring with resistance to macrolides and aminoglycosides [66]. Transfer of SCC*mec* from MRCoNS to methicillin‐sensitive *S. aureus* has been implicated in the evolution of new MRSA clones. The process relies on the SCC*mec* recombinase genes *ccrAB*, and the stability of the transferred SCC*mec* element varies depending on both the SCC*mec* type and the recipient strain [67]. Novel SCC*mec* cassettes carrying *fusC* and *tetK* genes responsible for particular drug resistance in environmental *Staphylococcus* species [68] (Table 2). A study on ready‐to‐eat foods in Maputo observed that both coagulase‐positive and CoNS strains harbored enterotoxin genes and drug resistance genes such as *blaZ*, *mecA*, and *vancA*. Among these strains, CoNS exhibited an exceptionally high rate of MDR, including vancomycin resistance, indicating that these harbor virulence factors and antimicrobial resistance genes (ARGs) [69]. Comparative overview of major CoNS species (*S. epidermidis*, *S. haemolyticus*, *S. saprophyticus*, *S. lugdunensis*), highlighting ecological niches, virulence factors, drug resistance mechanisms, and clinical significance (Table 3).

**Table 2:** Genes in the types of SCC*mec* responsible for resistance in environmental *Staphylococcus* species.

SCC*mec* type	*mec* gene complex	*ccr* gene complex	Characteristic resistance genes	Notable features
Type I (1B)	Class B	*ccrA1B1* (Type 1)	Inadequate resistance genes	Small cassette; mainly *β*‐lactam resistance; early hospital‐associated MRSA
Type II (2A)	Class A	*ccrA2B2* (Type 2)	Multiple resistance determinants in J regions	Large cassette; carries many additional resistance genes; classic HA‐MRSA.
Type III (3A)	Class A	*ccrA3B3* (Type 3)	Broad multidrug resistance genes	Very large SCCmec; hospital‐associated, prevalent in Asia and Oceania
Type IV (2B)	Class B	*ccrA2B2* (Type 2)	A few additional genes	Small, highly mobile; common in community‐associated MRSA (CA‐MRSA)
Type V (5C2)	Class C2	*ccrC* (Type 5)	Some additional resistance genes	Compact cassette; associated with livestock‐ and community‐associated strains

**Table 3:** Comparative overview of major coagulase‐negative staphylococcal (CoNS) species, highlighting ecological niches, virulence factors, drug resistance mechanisms, and clinical significance.

CoNS species	Primary ecological niche	Major virulence factors	Drug resistance genes/mechanisms	Clinical relevance	References
*Staphylococcus epidermidis*	Skin, mucous membranes, indwelling medical devices	Biofilm formation (icaADBC operon, PIA/PNAG), Aap, MSCRAMMs, extracellular DNA, phenol‐soluble modulins	*mecA/mecC*, *blaZ*, *erm*(*A/C*), *aac*(6 ^′^)‐*Ie-aph*(2 ^″^)*-Ia*, mutations in *gyrA/parC*	Major cause of catheter‐related bloodstream infections, prosthetic joint infections, neonatal sepsis, and other healthcare‐associated infections	Otto [14]; Becker et al. [70]; Heilmann et al. [71]
*Staphylococcus haemolyticus*	Skin, hospital environment	Biofilm formation, cytolysins, adhesins, extracellular enzymes	Frequent *mecA*, *blaZ*, *erm*, aminoglycoside‐modifying enzyme genes; high genomic plasticity and multidrug resistance	Important nosocomial pathogen causing bloodstream, wound, and device‐associated infections	Lamberte et al. [13]; Becker et al. [70]; Pain et al. [72], Rossi et al. [15]
*Staphylococcus saprophyticus*	Genitourinary tract, gastrointestinal tract, perineum	Urease, Aas and UafA adhesins, biofilm formation, D‐serine tolerance	Usually susceptible to many antibiotics; resistance by *blaZ*, *erm*, *tet* genes, and gyrase gene mutations	Second most common cause of uncomplicated urinary tract infection in young sexually active women	Becker et al. [70]; Kline & Lewis [73]
*Staphylococcus lugdunensis*	Skin (inguinal and perineal regions)	AtlL autolysin, fibrinogen‐binding proteins, hemolysins, biofilm formation	Generally antibiotic susceptible; occasional *mecA*, *blaZ*, and *erm* genes	Causes aggressive infections resembling those of *S. aureus*, including infective endocarditis, osteomyelitis, and prosthetic‐device infections	Heilbronner and Foster [74]; Becker et al. [70]; Frank et al. [75]

#### 4.1.2. HGT: Plasmids, Transposons, and Integrons

HGT is an important mechanism of the transmission of ARGs in CoNS and other bacterial communities [12]. Plasmids are highly mobile genetic elements that mediate the rapid exchange of ARGs that confer resistance to beta‐lactams, macrolides, tetracyclines, and aminoglycosides in CoNS [53, 54]. These genetic elements often include plasmids, transposons, and integrons that can harbor multiple antibiotic resistance genes simultaneously, which significantly enhances bacterial survival under selective pressure [59]. The plasmids harbor various resistance genes, namely, *blaZ* and *mecA* (showing resistance to *β*‐lactams), *ermC* (reported for resistance to macrolides, lincosamides, and streptogramin B), *cadA* (showing resistance to cadmium and zinc), *cadD* (showing resistance to cadmium), and *qacA* and *smr* (reported for resistance to biocides and dyes) [65, 76]. Transposons, specifically Tn3 and Tn4401, often harbor beta‐lactamase genes such as *blaTEM* and *blaKPC*. They can integrate into plasmids or the host chromosome [77, 78]. Tn554, carrying the *ermA* gene, mediates resistance to macrolide‐lincosamide‐streptogramin B (MLSB) antibiotics [55]. Transposons such as Tn916 and Tn1545 often carry the *tetM and ermB* genes, which confer resistance to tetracyclines and macrolides [79]. Class 1 integrons harboring *aadA*, *dfr*, and *cat* gene cassettes confer resistance to aminoglycosides, trimethoprim, and chloramphenicol, respectively [80]. In composite transposons, resistance genes are commonly bordered by insertion sequences, which enhance their mobility and facilitate stable integration into new genetic locations. Such cross‐species gene transfer events complicate treatment strategies and call for robust genetic surveillance [78]. Phenotypic and molecular mechanisms of AMR in CoNS are depicted in Figure 3.

**Figure 3:**
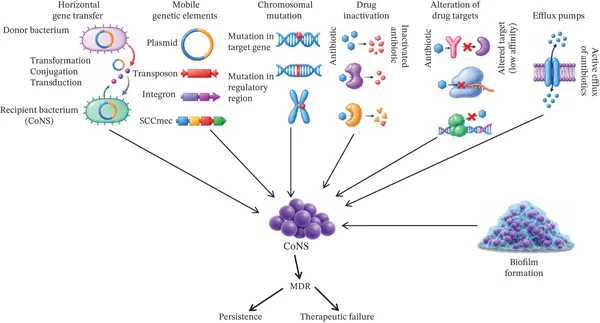
Phenotypic and molecular mechanisms of AMR in CoNS.

## 5. Biofilm Formation and Its Role in Resistance

### 5.1. Mechanisms of Biofilm Formation on Clinical and Environmental Surfaces

Biofilm is a key virulence trait of CoNS, which helps them survive adverse conditions, evade the immune system, and resist antimicrobial agents. The development of biofilms in CoNS, especially *S. epidermidis* and *S. haemolyticus*, has been reported and shown to involve stages: initial adhesion, biofilm accumulation and maturation, and subsequent detachment or dispersion [23]. The mechanism of adhesion involves the proteins of the surface of bacteria and extracellular DNA (eDNA), which play an important role in binding them to the surface of host proteins and implants. The largest constituent of the EPS matrix of CoNS biofilms is the polysaccharide intercellular adhesin (PIA), which is produced by the *icaADBC* operon [42, 81]. Recent studies have shown that clinical CoNS isolates have biofilm‐forming ability; approximately 90%–95% of isolates in the bloodstream form biofilms at the ends of catheters [43, 82]. Biofilm architecture facilitates nutrient gradients, cellular differentiation, and the development of persister cells that are vital under environmental stress and antimicrobial attack. The EPS matrix offers physical resistance to the penetration of antibiotics and disinfectants and serves as a trap in which enzymes break down the antimicrobials [83].

### 5.2. Biofilm Mediated Antibiotic Resistance and Novel Antibiofilm Strategies

CoNS infections are notoriously difficult to eliminate because of biofilms, which make them resistant to beta‐lactams, glycopeptides such as vancomycin, and aminoglycosides [44]. A few specific CoNS strains produce metabolites that can be employed for drug development against drug‐resistant microorganisms. Enhanced knowledge about CoNS behavior in food contamination may benefit industry efforts to develop contamination management strategies and food safety protocols [36, 84]. Research on CoNS could offer opportunities to develop probiotics and to create sustainable farming practices. Activities involving CoNS pose significant safety challenges, necessitating attention to antibiotic resistance and pathogenic behavior [85].

### 5.3. Combinatorial Therapy and Biofilm Disruption

In combinatorial therapies, antibiotics, along with biofilm‐disrupting agents, enzymes like dispersin B that degrade polysaccharides, or quorum‐sensing inhibitors, can weaken the biofilm matrix, which increased antibiotic penetration and bacterial susceptibility [86, 87]. Combinatorial treatments achieve higher biofilm eradication than antibiotics alone and reduce bacterial survival by 40%–70% in vitro [88]. Nanoparticle‐based drug delivery systems codeliver the antibiotics and antibiofilm agents, which enhance target‐based drug delivery and controlled release, and further improve treatment efficacy [89].

### 5.4. Bacteriophage‐Based Therapy

Bacteriophage therapy has potential against various multidrug‐resistant bacteria [90]. Phages can penetrate biofilms, lyse cells, and produce depolymerases that degrade EPS matrix components and directly disrupt biofilm development [91, 92]. Recent studies have developed phage cocktails by combining multiple phages targeting various types of bacteria, which effectively suppress bacterial growth and minimize resistance development [93]. Nanotechnology‐assisted phage delivery, such as phage‐loaded hydrogels, improves biofilm penetration and stability. Injectable hydrogels encapsulating phages targeting device infection biofilms show promise for localized therapy with controlled release [94].

### 5.5. Emerging Natural Products as Antimicrobial and Antibiofilm Agents

Novel antibiofilm agents, such as synthetic small molecules and natural product derivatives, target the pathways involved in biofilm formation or matrix components [95]. Cumarin derivatives, phenols, and furanones inhibit gene expression involved in adhesion and matrix synthesis, effectively preventing biofilm formation or inducing dispersal [56]. Natural and artificial AMPs and enzymes that degrade eDNA or polysaccharides represent additional emerging therapeutics. Some promising candidates exhibit synergistic activity with antibiotics in preclinical studies, although clinical translation requires further validation [56]. Phytochemicals exhibit promising antibacterial activities against CoNS. The minimum inhibitory concentrations (MICs) of the pellicle extract of *Juglans regia* (walnut) against CoNS ranged from 3.60 to 461.75 *μ*g/mL, as well as a minimum bactericidal concentration (MBC) of 461.75 *μ*g/mL. Moreover, the extract considerably inhibited biofilm formation and cell viability in a dose‐dependent manner, indicating that the flavonoid and the phenolic compounds could disrupt both bacterial development and biofilm formation [96]. Similarly, a polyhedral formula Ya‐Sa‐Marn‐Phlae (YSMP) and its constituent a‐mangostin produced by *Garcinia mangostana* were found to have potent antibacterial effects (MIC as low as 1–32 *μ*g/mL) against *Staphylococcus* species, including CoNS, and were able to prevent biofilm formation on both hydrophilic and hydrophobic surfaces [97]. Extracts of *Rosa canina*, *Rosa rugosa*, and *Rosa pomifera* ‘*Karpatia*’ exhibited pronounced antistaphylococcal activity against CoNS, with growth inhibition observed even at 1/4 MIC. SEM imaging showed membrane disintegration and cell lysis, which were confirmed as evidence of a phenolic‐rich Rosa extract is a promising plant‐derived antimicrobial. [98]. These results indicate that plant‐derived compounds may serve as natural substitutes for traditional antibiotics, particularly for treating biofilm‐related CoNS infections.

## 6. Advances in Diagnostic Methods and Surveillance Approaches

### 6.1. Modern Molecular and Genomic Approach

CoNS require biochemical resources to survive and adapt in diverse environments. Classical phenotypic tests like coagulase, catalase, and sugar fermentation profiles have partly been substituted with automated systems such as API Staph, Vitek 2, and Phoenix, which enhance diagnostic speed, robustness, and precision for identification of leading CoNS species like *S. epidermidis*, *S. saprophyticus*, and *S. haemolyticus* [2, 99]. Although these systems tend to perform poorly with rare or animal‐origin isolates, highlighting the importance of molecular approaches facilitating reliable species differentiation, particularly in nonclinical environmental studies [1] (Figure 4). Genomic studies emphasize the vast genetic heterogeneity of CoNS, whereas *S. aureus* has long dominated staphylococci. These studies indicate widespread genome flexibility of *S. epidermidis*, *S. haemolyticus*, and *S. capitis*, fueled by mobile genetic elements like plasmids, insertion sequences, and the staphylococcal cassette chromosome *mec* (SCC*mec*), which allow their quick adaptation to antibiotic pressure and environmental stress [14, 17, 51]. CRISPR‐based approaches have gained significant attention for analyzing ARG functions and pathways. High‐throughput imaging, including electron and fluorescence microscopy, plays a vital role in studying biofilm structures and microbial interactions in complex nonclinical systems. Screening by whole‐genome sequencing (WGS) enables tracing and analysis of CoNS populations across dry soil, liquid environments, and food‐industrial environments [57]. The utilization of AI and ML technology yields substantial benefits for microbial research operations. These technologies are used to handle large datasets to identify CoNS behavioral patterns across population groups and to forecast trends in resistance development. Technologies that monitor real‐time environmental data, coupled with recent advances, enable better management of CoNS challenges arising from persistence, resistance dynamics, and transmission patterns [100]. Activities involving CoNS pose significant safety challenges, necessitating attention to antibiotic resistance and pathogenic behavior. Safety, along with the efficient implementation of the antibiotic system in scientific applications, requires further research and greater attention from government bodies.

**Figure 4:**
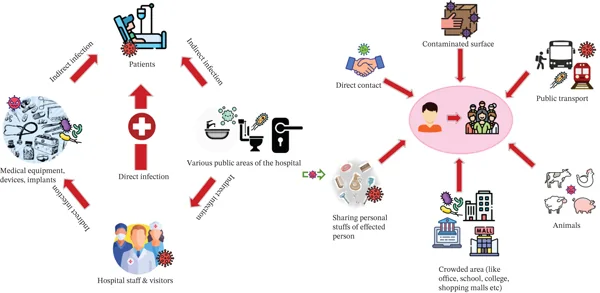
The spread of antimicrobial‐resistant (AMR) CoNS bacteria from regions with extensive antibiotic use to areas with minimal antibiotic use, as well as their transmission to humans in public environments.

### 6.2. WGS and Molecular Typing

Advancements in WGS and molecular typing have transformed the understanding of resistance acquisition. WGS provides high‐resolution data on genetic determinants, mobile elements, mutation rates, and phylogenetic relationships, enabling precise tracking of resistance gene flow within and between populations. WGS and first‐line molecular typing (e.g., MLST, PFGE) can be combined to balance costs and resolution to support epidemiological surveillance [101]. WGS provided fine‐scale resolution of the *Staphylococcus warneri* NICU outbreak, distinguishing the sporadic 2020 isolate from the clonal 2021 isolates using core‐genome SNPs. It also established transmission links between neonatal and environmental isolates, identified resistance determinants (*mecA, bleO, dfrG*), and validated shared virulence profiles. With this genomic resolution, attribution of the source and targeted interventions of infection control became straightforward [102]. Overall, these molecular epidemiological methods are valuable tools for mapping the spread and movement of CoNS in different nonclinical settings. Through these approaches, researchers can identify probable sources and routes of bacterial transmission in agricultural settings, food processing environments, and high public contact places [103].

## 7. One Health Approach and Public Health Implications

The One Health approach highlights the interdependence of animal, human, and environmental health, which requires a renewed emphasis on these environmental reservoirs of virulence and resistance, where CoNS can evolve, adapt, and spread beyond the clinic [60]. The sharing of *mecA* and SCC*mec* between livestock and human staphylococci from a One Health perspective showed SCC*mec* type V (MRSA CC398), together with *tetM* (tetracycline resistance), *ermB* (macrolide resistance), and *czrC* (zinc resistance), and recurrent deletions in human immune evasion genes, such as *scn, chp,* and *sak*, while also demonstrating an enrichment of lactose operons, such as *lacA* and *lacB*, conferring a competitive advantage in cow milk. WGS of bovine milk has revealed many dynamic accessory genomes, with the presence of *blaZ* beta‐lactamase and *tetK* located on the prophages. Indian mastitis cases display a fully open pan‐genome, including large numbers of plasmids and ongoing HGT from these cases. [12]. Similar data from CoNS, such as *S. chromogenes* identified in cattle, have shown the presence of *tetK* and *ermC* on mobile genetic elements and are considered similar to multidrug‐resistant profiles in humans. Therefore, farms disseminate ARGs through the use of manure, dust, and wastewater [103].

## 8. Government–Public–Private Collaboration

CoNS research investigates their persistence under diverse environmental conditions and clinical settings. Restricting the use of antibiotics in veterinary and agricultural settings is a significant factor in preventing the emergence of antibiotic‐resistant CoNS [9]. Antibiotic stewardship programs, coupled with alternative measures such as improved sanitation, vaccination schemes, and stringent biosecurity measures, could reduce the development of resistant strains and their spread in the environment [104]. Addressing the persistence and spread of CoNS requires an organized effort of government, public health, private‐sector partners, the agricultural sector, veterinary medicine, and environmental management. Successful collaboration involves the sharing of surveillance data, research findings, and operational strategies to combat resistance and inhibit the spread [105]. Raising awareness among the major stakeholders, farmers, food industry workers, healthcare workers, and the public about the consequences of CoNS and antibiotic resistance is crucial. Educating the public on optimal hygiene, food handling, and antimicrobial use can improve public and environmental health outcomes.

## 9. Knowledge Gaps and Future Perspectives

Despite pronounced recognition of CoNS as environmentally and clinically meaningful reservoirs of AMR, imperative knowledge gaps remain: (i) heterogeneous epidemiological surveillance across geographic areas and nonclinical and clinical settings, restricting exact determination of the global load of CoNS resistance and infections; (ii) distinguishing true infection, contamination, and colonization remains a consistent diagnostic hurdle; (iii) the contribution of animal‐ and environment‐linked CoNS to the dissemination and acquisition of clinically imperative AMR factors needs further assessment. Genomic monitoring incorporating epidemiological data with WGS may enhance the detection of transmission routes and AMR gene flow. Further, investigations are also needed to explain the molecular interactions between persistence, biofilm formation, and antimicrobial tolerance. Furthermore, antimicrobial combinations, bacteriophage‐driven strategies, novel antibiofilm drugs, and other complementary therapies need systematic assessment in clinically meaningful models prior to translation into routine clinical practice. A collaborative “One Health surveillance” system combining environmental, human, food, and animal repertoires will be imperative for ameliorating these hurdles.

## 10. Conclusion

CoNS have become important pathogens in clinical practice, particularly in machine‐related infections, neonatal sepsis, and BSIs, a trend exacerbated by widespread AMR. Their widespread presence in various environmental reservoirs, such as soil, water, livestock, and common areas, provides a venue for the exchange and distribution of resistance genes. A *mecA* gene located between multiple *SCCmec* elements provides 2‐lactam resistance and is the key determinant of methicillin resistance in CoNS. The possible transfer of many resistance loci via HGT mediated by plasmids, transposons, and integrases enables the rapid dissemination of these loci. CoNS pathogenicity is a characteristic feature of biofilm development, enabling their adherence to medical devices and the persistence of infections. There is a glimmer of hope that novel antibiofilm interventions—combinatorial antibiotic regimens, bacteriophage therapy, and new antibiofilm agents can be used in order to overcome biofilm‐associated resistance. The CoNS AMR challenge cannot be addressed without a One Health framework, which recognizes the relationship between human, animal, and environmental health. Integrated educational programs across sectors, coordinated surveillance, antimicrobial stewardship activities, government partnerships with industry, and other efforts are essential to check the proliferation of resistant CoNS. The AMR threat posed by CoNS requires ongoing, cross‐disciplinary cooperation to support surveillance systems and implement evidence‐based measures locally, nationally, and internationally.

## Author Contributions

Divakar Sharma conceived the idea. Anushri Keshri, Shivani Choudhary, Anil Kumar, Indu Singh, Haritima Singh, Divakar Sharma, Arun Kumar, Sushma Verma, and Arun Ratn wrote the initial draft and prepared the figures. Divakar Sharma extensively edited the manuscript. All authors reviewed the final manuscript. Anushri Keshri and Shivani Choudhary contributed equally.

## Funding

No funding was received for this manuscript.

## Ethics Statement

The authors have nothing to report.

## Consent

The authors have nothing to report.

## Conflicts of Interest

The authors declare no conflicts of interest.

## Data Availability

Data sharing is not applicable to this article, as no datasets were generated or analyzed during the current study.

## References

[1] Patil G. , Agarwala P. , Das P. , and Pathak S. , Rise in the Pathogenic Status of Coagulase-Negative Staphylococci Causing Bloodstream Infection, Cureus. (2024) 16, no. 3, e57250, 10.7759/cureus.57250, 38686262. PMC11056806 38686262

[2] Adesoji T. O. , Somda N. S. , Tetteh-Quarcoo P. , Shittu A. O. , and Donkor E. S. , Prevalence of Methicillin-Resistant Coagulase-Negative Staphylococci in Africa: A Systematic Review and Meta-Analysis, BMC Infectious Diseases. (2025) 25, no. 1, 10.1186/s12879-025-11149-1, 40640717. PMC12243303 40640717

[3] Byrd A. L. , Belkaid Y. , and Segre J. A. , The Human Skin Microbiome, Nature Reviews Microbiology. (2018) 16, no. 3, 143–155, 10.1038/nrmicro.2017.157. 29332945

[4] Severn M. M. and Horswill A. R. , *Staphylococcus epidermidis* and Its Dual Lifestyle in Skin Health and Infection, Nature Reviews Microbiology. (2023) 21, no. 2, 97–111, 10.1038/s41579-022-00780-3, 36042296. PMC9903335 36042296

[5] Philip N. , Suneja B. , and Walsh L. , Beyond Streptococcus mutans: Clinical Implications of the Evolving Dental Caries Aetiological Paradigms and Its Associated Microbiome, British Dental Journal. (2018) 224, no. 4, 219–225, 10.1038/sj.bdj.2018.81, 29449651. 29449651

[6] Dadi N. C. T. , Radochová B. , Vargová J. , and Bujdáková H. , Impact of Healthcare-Associated Infections Connected to Medical Devices—An update, Microorganisms. (2021) 9, no. 11, 10.3390/microorganisms9112332, 34835457. PMC8618630 34835457

[7] Lazar V. , Badaluta V.-A. , Apetrei R. M. , and Ditu L.-M. , *Staphylococcus aureus*—A Quiet Human Body Resident and Sometimes a Feared Enemy, Journal of Microbes in Health and Disease. (2025) 1, no. 1, https://www.sciltp.com/journals/jmhd/articles/2505000639, 100002.

[8] Bouhrour N. , Nibbering P. H. , and Bendali F. , Medical Device-Associated Biofilm Infections and Multidrug-Resistant Pathogens, Pathogens. (2024) 13, no. 5, 10.3390/pathogens13050393, 38787246. PMC11124157 38787246

[9] França A. , Gaio V. , Lopes N. , and Melo L. D. , Virulence Factors in Coagulase-Negative Staphylococci, Pathogens. (2021) 10, no. 2, 10.3390/pathogens10020170, 33557202. PMC7913919 33557202

[10] Wang B. , Li M. , Yu J. , Jiang F. , Wang C. , Hu Y. , Tang J. , and Shen H. , Global Prevalence and Antimicrobial Resistance of Staphylococcus aureus Isolates From Patients With Musculoskeletal Infections: A Systematic Review and Meta-Analysis, International Journal of Surgery. (2026) 112, no. 2, 4981–4989, 10.1097/JS9.0000000000003862, 41731880. 41731880

[11] Ribeiro A. C. , Alves D. T. , Campos G. Z. , Da Costa T. G. , de Melo Franco B. D. G. , de Almeida F. A. , and Pinto U. M. , Clarifying the Dual Role of Staphylococcus spp. in Cheese Production, Foods. (2025) 14, no. 22, 10.3390/foods14223823. PMC12651221 41299981

[12] Elbehiry A. and Marzouk E. , Staphylococci in Livestock: Molecular Epidemiology, Antimicrobial Resistance, and Translational Strategies for One Health Protection, Veterinary Sciences. (2025) 12, no. 8, 10.3390/vetsci12080757, 40872707. PMC12390266 40872707

[13] Lamberte L. E. , Darby E. M. , Kiu R. , Moran R. A. , Acuna-Gonzalez A. , Sim K. , Shaw A. G. , Kroll J. S. , Belteki G. , Clarke P. , and Felgate H. , *Staphylococcus haemolyticus* is a Reservoir of Antibiotic Resistance Genes in the Preterm Infant Gut, Gut Microbes. (2025) 17, no. 1, 2519700, 10.1080/19490976.2025.2519700, 40544427. PMC12323770 40544427

[14] Otto M. , *Staphylococcus epidermidis*—The ‘Accidental’ Pathogen, Nature Reviews Microbiology. (2009) 7, no. 8, 555–567, 10.1038/nrmicro2182, 19609257. PMC2807625 19609257

[15] Rossi C. C. , Ahmad F. , and Giambiagi-deMarval M. , *Staphylococcus haemolyticus*: An Updated Review on Nosocomial Infections, Antimicrobial Resistance, Virulence, Genetic Traits, and Strategies for Combating This Emerging Opportunistic Pathogen, Microbiological Research. (2024) 282, 127652, 10.1016/j.micres.2024.127652, 38432015. 38432015

[16] Cole K. , Atkins B. , Llewelyn M. , and Paul J. , Genomic Investigation of Clinically Significant Coagulase-Negative Staphylococci, Journal of Medical Microbiology. (2021) 70, no. 3, 001337, 10.1099/jmm.0.001337. 33704043

[17] Foster T. J. , Antibiotic resistance in *Staphylococcus aureus*. Current Status and Future Prospects, FEMS Microbiology Reviews. (2017) 41, no. 3, 430–449, 10.1093/femsre/fux007, 28419231. 28419231

[18] Eltwisy H. O. , Twisy H. O. , Hafez M. H. , Sayed I. M. , and El-Mokhtar M. A. , Clinical Infections, Antibiotic Resistance, and Pathogenesis of Staphylococcus haemolyticus, Microorganisms. (2022) 10, no. 6, 10.3390/microorganisms10061130, 35744647. PMC9231169 35744647

[19] Naing S. Y. , Duim B. , Broens E. M. , Schweitzer V. , Zomer A. , van der Graaf-Van Bloois L. , Van Der Meer C. , Stellingwerff L. , Fluit A. C. , and Wagenaar J. A. , Molecular Characterization and Clinical Relevance of Taxonomic Reassignment of *Staphylococcus schleiferi* Subspecies Into Two Separate Species, *Staphylococcus schleiferi* and *Staphylococcus coagulans* , Microbiology Spectrum. (2023) 11, no. 2, e04670-22, 10.1128/spectrum.04670-22, 36853031. PMC10101015 36853031

[20] Ahle C. M. , Geno-and Phenotypic Characterisation of Coagulase-Negative Staphylococci on Healthy Human Skin, 2022, University of Hamburg, https://ediss.sub.uni-hamburg.de/handle/ediss/9717.

[21] Goetz C. , Tremblay Y. D. , Lamarche D. , Blondeau A. , Gaudreau A. M. , Labrie J. , Malouin F. , and Jacques M. , Coagulase-Negative Staphylococci Species Affect Biofilm Formation of Other Coagulase-Negative and Coagulase-Positive Staphylococci, Journal of Dairy Science. (2017) 100, no. 8, 6454–6464, 10.3168/jds.2017-12629, 28624271. 28624271

[22] Paharik A. E. and Horswill A. R. , The Staphylococcal Biofilm: Adhesins, Regulation, and Host Response, Virulence Mechanisms of Bacterial Pathogens. (2016) 4, 529–566, 10.1128/microbiolspec.vmbf-0022-2015. PMC4887152 27227309

[23] Fredheim E. G. A. , Klingenberg C. , Rohde H. , Frankenberger S. , Gaustad P. , Flægstad T. , and Sollid J. E. , Biofilm formation by Staphylococcus haemolyticus, Journal of Clinical Microbiology. (2009) 47, no. 4, 1172–1180, 10.1128/JCM.01891-08, 19144798. PMC2668337 19144798

[24] Lin L.-C. , Shu J.-C. , Chang S.-C. , Ge M.-C. , Liu T.-P. , Chen C.-W. , and Lu J.-J. , Nucleotide Sequence Variations in Autolysis Genes of ST59 Methicillin-Resistant *Staphylococcus aureus* Isolates, Microbial Drug Resistance. (2017) 23, no. 8, 940–948, 10.1089/mdr.2016.0098, 28358616. 28358616

[25] Kresken M. , Becker K. , Seifert H. , Leitner E. , Körber-Irrgang B. , Von Eiff C. , Löschmann P.-A. , and Study Group , Resistance Trends and In Vitro Activity of Tigecycline and 17 Other Antimicrobial Agents Against Gram-Positive and Gram-Negative Organisms, Including Multidrug-Resistant Pathogens, in Germany, European Journal of Clinical Microbiology & Infectious Diseases. (2011) 30, no. 9, 1095–1103, 10.1007/s10096-011-1197-y, 21347680. 21347680

[26] Di Francesco A. , Mari M. , Ugolini L. , and Baraldi E. , Effect of *Aureobasidium pullulans* Strains Against *Botrytis cinerea* on Kiwifruit During Storage and on Fruit Nutritional Composition, Food Microbiology. (2018) 72, 67–72, 10.1016/j.fm.2017.11.010. 29407406

[27] Kiedrowski M. R. and Horswill A. R. , New Approaches for Treating Staphylococcal Biofilm Infections, Annals of the New York Academy of Sciences. (2011) 1241, no. 1, 104–121, 10.1111/j.1749-6632.2011.06281.x. 22191529

[28] Keshri A. , Gore D. G. , Singh I. , Sharma D. , and Kolla V. , Molecular Characterization of Cefoxitin-Resistant Coagulase-Negative Staphylococci From Frequently Touched Surfaces of Hospital and Urban-Built Environments of Central India, Canadian Journal of Infectious Diseases and Medical Microbiology. (2025) 2025, no. 1, 5766823, 10.1155/cjid/5766823, 39949528. PMC11824842 39949528

[29] Xu Z. , Liu S. , Chen L. , Liu Y. , Tan L. , Shen J. , and Zhang W. , Antimicrobial Resistance and Molecular Characterization of Methicillin-Resistant Coagulase-Negative Staphylococci From Public Shared Bicycles in Tianjin, China, Journal of Global Antimicrobial Resistance. (2019) 19, 231–235, 10.1016/j.jgar.2019.03.008, 30910743. 30910743

[30] Vink J. , Edgeworth J. , and Bailey S. L. , Acquisition of MDR-GNB in Hospital Settings: A Systematic Review and Meta-Analysis Focusing on ESBL-E, Journal of Hospital Infection. (2020) 106, no. 3, 419–428, 10.1016/j.jhin.2020.09.006. 32918969

[31] Heo S. , Lee J.-H. , and Jeong D.-W. , Food-Derived Coagulase-Negative *Staphylococcus* as Starter Cultures for Fermented Foods, Food Science and Biotechnology. (2020) 29, no. 8, 1023–1035, 10.1007/s10068-020-00789-5, 32670656. PMC7347722 32670656

[32] Khalifa A. A. , Bearded Surgeons: Friend or Foe in the Fight Against Surgical Site Infections? An Updated and Comprehensive Literature Review, Journal of Musculoskeletal Surgery and Research. (2025) 9, no. 3, 310–316, 10.25259/JMSR_148_2025.

[33] Mkrtchyan H. V. , Russell C. A. , Wang N. , and Cutler R. R. , Could Public Restrooms be an Environment for Bacterial Resistomes?, PloS One. (2013) 8, no. 1, e54223, 10.1371/journal.pone.0054223, 23349833. PMC3547874 23349833

[34] Keshri A. and Kolla V. , Comparative Antibiotic Susceptibility Profiling of S. *haemolyticus* Recovered From Frequency Hand-Touched Surfaces of Hospital Settings and Urban Built Environments of Central India, NewBioWorld. (2024) 6, no. 2, 1–7, 10.52228/NBW-JAAB.2024-6-2-1.

[35] Arciola C. R. , Campoccia D. , Speziale P. , Montanaro L. , and Costerton J. W. , Biofilm Formation in Staphylococcus Implant Infections. A Review of Molecular Mechanisms and Implications for Biofilm-Resistant Materials, Biomaterials. (2012) 33, no. 26, 5967–5982, 10.1016/j.biomaterials.2012.05.031, 22695065. 22695065

[36] França A. , The Role of Coagulase-Negative Staphylococci Biofilms on Late-Onset Sepsis: Current Challenges and Emerging Diagnostics and Therapies, Antibiotics. (2023) 12, no. 3, 10.3390/antibiotics12030554. PMC10044083 36978421

[37] Burke Ó. , Zeden M. S. , and O’Gara J. P. , The Pathogenicity and Virulence of the Opportunistic Pathogen *Staphylococcus epidermidis* , Virulence. (2024) 15, no. 1, 2359483, 10.1080/21505594.2024.2359483. PMC11178275 38868991

[38] OKeefe K. , Diversity, Genomics and Antimicrobial Resistance of Coagulase-Negative Staphylococcus: A Literature Review, 2025, University at Albany, State University of New York, https://scholarsarchive.library.albany.edu/cgi/viewcontent.cgi?article=1115%26context=etd.

[39] Jansen S. J. , Broer S. D. , Hemels M. A. , Visser D. H. , Antonius T. A. , Heijting I. E. , Bergman K. A. , Termote J. U. , Hütten M. C. , van der Sluijs J. P. , d’Haens E. J. , Kornelisse R. F. , Lopriore E. , and Bekker V. , Central-Line-Associated Bloodstream Infection Burden Among Dutch Neonatal Intensive care Units, Journal of Hospital Infection. (2024) 144, no. 144, 20–27, 10.1016/j.jhin.2023.11.020. 38103692

[40] Scamardo M. S. , Dolce P. , Esposito E. P. , Raimondi F. , Triassi M. , and Zarrilli R. , Trends, Risk Factors and Outcomes of Healthcare-Associated Infections in a Neonatal Intensive Care Unit in Italy During 2013–2017, Italian Journal of Pediatrics. (2020) 46, no. 1, 10.1186/s13052-020-0799-3, 32183842. PMC7079437 32183842

[41] Liu S. , Chen H. , Xu F. , Chen F. , Yin Y. , Zhang X. , Tu S. , and Wang H. , Unravelling Staphylococcal Small-Colony Variants in Cardiac Implantable Electronic Device Infections: Clinical Characteristics, Management, and Genomic Insights, Frontiers in Cellular and Infection Microbiology. (2024) 13, 1321626, 10.3389/fcimb.2023.1321626, 38259974. PMC10800868 38259974

[42] François P. , Schrenzel J. , and Götz F. , Biology and Regulation of Staphylococcal Biofilm, International Journal of Molecular Sciences. (2023) 24, no. 6, 10.3390/ijms24065218, 36982293. PMC10049468 36982293

[43] Grassia G. , Bagnarino J. , Siciliano M. , Barbarini D. , Corbella M. , Cambieri P. , Baldanti F. , and Monzillo V. , Phenotypic and Genotypic Assays to Evaluate Coagulase-Negative Staphylococci Biofilm Production in Bloodstream Infections, Microorganisms. (2024) 12, no. 1, 10.3390/microorganisms12010126, 38257953. PMC10818815 38257953

[44] Kranjec C. , Morales Angeles D. , Torrissen Mårli M. , Fernández L. , García P. , Kjos M. , and Diep D. B. , Staphylococcal Biofilms: Challenges and Novel Therapeutic Perspectives, Antibiotics. (2021) 10, no. 2, 10.3390/antibiotics10020131, 33573022. PMC7911828 33573022

[45] Bizzarro M. J. , Shabanova V. , Baltimore R. S. , Dembry L.-M. , Ehrenkranz R. A. , and Gallagher P. G. , Neonatal Sepsis 2004-2013: The Rise and Fall Of Coagulase-Negative Staphylococci, Journal of Pediatrics. (2015) 166, no. 5, 1193–1199, 10.1016/j.jpeds.2015.02.009, 25919728. PMC4413005 25919728

[46] Bora P. , Datta P. , Gupta V. , Singhal L. , and Chander J. , Characterization and Antimicrobial Susceptibility of Coagulase-Negative Staphylococci Isolated From Clinical Samples, Journal of Laboratory Physicians. (2018) 10, no. 4, 414–419, 10.4103/JLP.JLP_55_18, 30498314. PMC6210844 30498314

[47] Marincola G. , Liong O. , Schoen C. , Abouelfetouh A. , Hamdy A. , Wencker F. D. , Marciniak T. , Becker K. , Köck R. , and Ziebuhr W. , Antimicrobial Resistance Profiles of Coagulase-Negative Staphylococci in Community-Based Healthy Individuals in Germany, Frontiers in Public Health. (2021) 9, 684456, 10.3389/fpubh.2021.684456, 34222184. PMC8247762 34222184

[48] Cave R. , Misra R. , Chen J. , Wang S. , and Mkrtchyan H. V. , Whole Genome Sequencing Revealed New Molecular Characteristics in Multidrug Resistant Staphylococci Recovered From High Frequency Touched Surfaces in London, Scientific Reports. (2019) 9, no. 1, 10.1038/s41598-019-45886-6, 31371820. PMC6675788 31371820

[49] Morales-Ubaldo A. L. , Rivero-Perez N. , Valladares-Carranza B. , Velázquez-Ordoñez V. , Delgadillo-Ruiz L. , and Zaragoza-Bastida A. , Bovine Mastitis, a Worldwide Impact Disease: Prevalence, Antimicrobial Resistance, and Viable Alternative Approaches, Veterinary and Animal Science. (2023) 21, 100306, 10.1016/j.vas.2023.100306, 37547227. PMC10400929 37547227

[50] Yang Y. , Sun Y. , Zhou Z. , Song Y. , Zhu Y. , Zhou W. , Yue M. , Zhao G. , Jiang H. , and Tang B. , Surveillance of Escherichia coli Antimicrobial Resistance in Pig Farms in Zhejiang Province, China: High Prevalence of Multidrug Resistance and Risk-Associated Genes, Microbial Pathogenesis. (2025) 204, 107598, 10.1016/j.micpath.2025.107598, 40250499. 40250499

[51] Wolska-Gębarzewska M. , Międzobrodzki J. , and Kosecka-Strojek M. , Current Types of Staphylococcal Cassette Chromosome mec (SCC mec) in Clinically Relevant Coagulase-Negative Staphylococcal (CoNS) species, CritiCal Reviews in MiCrobiology. (2024) 50, no. 6, 1020–1036, 10.1080/1040841X.2023.2274841. 37882662

[52] Shrestha L. B. , Bhattarai N. R. , and Khanal B. , Antibiotic Resistance and Biofilm Formation Among Coagulase-Negative Staphylococci Isolated From Clinical Samples at a Tertiary Care Hospital of Eastern Nepal, Antimicrobial Resistance & Infection Control. (2017) 6, no. 1, 10.1186/s13756-017-0251-7, 28883911. PMC5579930 28883911

[53] Fišarová L. , Pantůček R. , Botka T. , and Doškař J. , Variability of Resistance Plasmids in Coagulase-Negative Staphylococci and Their Importance as a Reservoir of Antimicrobial Resistance, Research in Microbiology. (2019) 170, no. 2, 105–111, 10.1016/j.resmic.2018.11.004, 30503569. 30503569

[54] Sabzehali F. , Goudarzi M. , Goudarzi H. , and Azimi H. , Distribution of Aminoglycoside Resistance Genes in Coagulase-Negative Staphylococci Isolated From Hospitalized Patients, Arch Pediatr Infect Dis. (2017) 5, no. 3, 10.3390/pathogens15070745, 42515072.

[55] Khodabandeh M. , Mohammadi M. , Abdolsalehi M. R. , Alvandimanesh A. , Gholami M. , Bibalan M. H. , Pournajaf A. , Kafshgari R. , and Rajabnia R. , Analysis of Resistance to Macrolide–Lincosamide–Streptogramin B Among mecA-Positive *Staphylococcus aureus* Isolates, Osong Public Health and Research Perspectives. (2019) 10, no. 1, 25–31, 10.24171/j.phrp.2019.10.1.06, 30847268. PMC6396818 30847268

[56] Ahmed G. E. , Elshahid Z. A. , El-Sawy E. R. , Abdel-Aziz M. S. , and Abdel-Aziem A. , Synthesis, Biofilm Formation Inhibitory, and Inflammation Inhibitory Activities of New Coumarin Derivatives, Scientific Reports. (2024) 14, no. 1, 10.1038/s41598-024-59072-w, 38643226. PMC11032357 38643226

[57] Coppola F. , Fratianni F. , Bianco V. , Wang Z. , Pellegrini M. , Coppola R. , and Nazzaro F. , New Methodologies as Opportunities in the Study of Bacterial Biofilms, Including Food-Related Applications, Microorganisms. (2025) 13, no. 5, 10.3390/microorganisms13051062. PMC12114119 40431235

[58] Ito T. , Hiramatsu K. , Tomasz A. , de Lencastre H. , Perreten V. , Holden M. , Coleman D. , Goering R. , Giffard P. , Skov R. , Zhang K. , Westh H. , O′Brien F. , Tenover F. C. , Oliveira D. C. , Boyle-Vavra S. , Laurent F. , Kearns A. M. , Kreiswirth B. , Ko K. S. , Grundmann H. , Sollid J. E. , John JF Jr , Daum R. , Soderquist B. , Buist G. , and International Working Group on the Classification of Staphylococcal Cassette Chromosome Elements (IWG-SCC) , Guidelines for Reporting Novel *mecA* Gene Homologues, Antimicrobial Agents and Chemotherapy. (2012) 56, no. 10, 4997–4999, 10.1128/AAC.01199-12, 22869575. PMC3457410 22869575

[59] Lerminiaux N. A. and Cameron A. D. , Horizontal Transfer of Antibiotic Resistance Genes in Clinical Environments, Canadian Journal of Microbiology. (2019) 65, no. 1, 34–44, 10.1139/cjm-2018-0275. 30248271

[60] Haworth-Brockman M. , Saxinger L. M. , Miazga-Rodriguez M. , Wierzbowski A. , and Otto S. J. , One Health Evaluation of Antimicrobial Use and Resistance Surveillance: A Novel Tool for Evaluating Integrated, One Health Antimicrobial Resistance and Antimicrobial Use Surveillance Programs, Frontiers in Public Health. (2021) 9, 693703, 10.3389/fpubh.2021.693703, 34422748. PMC8371385 34422748

[61] International Working Group on the Staphylococcal Cassette Chromosome , Classification of Staphylococcal Cassette Chromosome mec (SCCmec): Guidelines for Reporting Novel SCCmec Elements, Antimicrobial Agents and Chemotherapy. (2009) 53, no. 12, 4961–4967, 10.1128/AAC.00579-09, 19721075. PMC2786320 19721075

[62] Panel O. H. H.-L. E. , Hayman D. T. , Adisasmito W. B. , Almuhairi S. , Behravesh C. B. , Bilivogui P. , Bukachi S. A. , Casas N. , Becerra N. C. , and Charron D. F. , Developing One Health Surveillance Systems, One Health. (2023) 17, 100617, 10.1016/j.onehlt.2023.100617. PMC10665171 38024258

[63] Narayanan S. , Speciation of Clinically Significant Coagulase Negative Staphylococcus (CoNs) & Detection of Reduced Susceptibility of Vancomycin Among Methicillin Resistant-CoNs, 2020, Rajiv Gandhi University of Health Sciences, https://www.proquest.com/openview/e79946271191e4e8e0fb53387a73bf73/1?pq-origsite=gscholar%26cbl=2026366%26diss=y.

[64] Peng X. , Zhu Q. , Liu J. , Zeng M. , Qiu Y. , Zhu C. , Cheng Y. , Zhou Y. , Xu Y. , and Chen M. , Prevalence and Antimicrobial Resistance Patterns of Bacteria Isolated From Cerebrospinal Fluid Among Children With Bacterial Meningitis in China from 2016 to 2018: A Multicenter Retrospective Study, Antimicrobial Resistance & Infection Control. (2021) 10, no. 1, 24, 10.1186/s13756-021-00895-x, 33516275. PMC7847565 33516275

[65] Hanssen A.-M. and Ericson Sollid J. U. , SCCmecin Staphylococci: Genes on the Move, FEMS Immunology & Medical Microbiology. (2006) 46, no. 1, 8–20, 10.1111/j.1574-695X.2005.00009.x, 16420592. 16420592

[66] Szemraj M. , Glajzner P. , and Sienkiewicz M. , Decreased Susceptibility to Vancomycin and Other Mechanisms of Resistance to Antibiotics in *Staphylococcus epidermidis* as a Therapeutic Problem in Hospital Treatment, Scientific Reports. (2023) 13, no. 1, 13629, 10.1038/s41598-023-40866-3, 37604965. PMC10442409 37604965

[67] Maree M. , Thi Nguyen L. T. , Ohniwa R. L. , Higashide M. , Msadek T. , and Morikawa K. , Natural Transformation Allows Transfer of SCC mec-Mediated Methicillin Resistance in *Staphylococcus aureus* Biofilms, Nature Communications. (2022) 13, no. 1, 2477, 10.1038/s41467-022-29877-2, 35513365. PMC9072672 35513365

[68] El-Deeb W. , Cave R. , Fayez M. , Alhumam N. , Quadri S. , and Mkrtchyan H. V. , Methicillin-Resistant Staphylococci Isolated From Goats and Their Farm Environments in Saudi Arabia Genotypically Linked to Known Human Clinical Isolates: A Pilot Study, Microbiology Spectrum. (2022) 10, no. 4, e00387-22, 10.1128/spectrum.00387-22, 35913203. PMC9431424 35913203

[69] Salamandane A. , Oliveira J. , Coelho M. , Ramos B. , Cunha M. V. , Malfeito-Ferreira M. , and Brito L. , Enterotoxin-and Antibiotic-Resistance-Encoding Genes Are Present in Both Coagulase-Positive and Coagulase-Negative Foodborne *Staphylococcus* Strains, Applied Microbiology. (2022) 2, no. 2, 367–380, 10.3390/applmicrobiol2020028.

[70] Becker K. , Heilmann C. , and Peters G. , Coagulase-Negative Staphylococci, Clinical Microbiology Reviews. (2014) 27, no. 4, 870–926, 10.1128/CMR.00109-13. PMC4187637 25278577

[71] Heilmann C. , Ziebuhr W. , and Becker K. , Are Coagulase-Negative Staphylococci Virulent?, Clinical Microbiology and Infection. (2019) 25, no. 9, 1071–1080, 10.1016/j.cmi.2018.11.012. 30502487

[72] Pain M. , Hjerde E. , Klingenberg C. , and Cavanagh J. P. , Comparative Genomic Analysis of *Staphylococcus haemolyticus* Reveals Key to Hospital Adaptation and Pathogenicity, Frontiers in Microbiology.(2019) 10, 10.3389/fmicb.2019.02096. PMC6747052 31552006

[73] Kline K. A. and Lewis A. L. , Gram-Positive Uropathogens, Polymicrobial Urinary Tract Infection, and the Emerging Microbiota of the Urinary Tract, Microbiology Spectrum. (2016) 4, no. 2, 10.1128/microbiolspec.UTI-0012-2012, 27227294. PMC4888879 27227294

[74] Heilbronner S. and Foster T. J. , *Staphylococcus lugdunensis*: A Skin Commensal With Invasive Pathogenic Potential, Clinical Microbiology Reviews. (2021) 34, no. 2, 10–1128, 10.1128/CMR.00205-20. PMC7950365 33361142

[75] Frank K. L. , Del Pozo J. L. , and Patel R. , From Clinical Microbiology to Infection Pathogenesis: How Daring Is *Staphylococcus lugdunensis*?, Clinical Microbiology Reviews. (2008) 21, no. 1, 111–133, 10.1128/CMR.00036-07, 18202439. PMC2223846 18202439

[76] Costa S. S. , Palma C. , Kadlec K. , Fessler A. T. , Viveiros M. , Melo-Cristino J. , Schwarz S. , and Couto I. , Plasmid-Borne Antimicrobial Resistance ofStaphylococcus aureusIsolated in a Hospital in Lisbon, Portugal, Microbial Drug Resistance. (2016) 22, no. 8, 617–626, 10.1089/mdr.2015.0352, 27309487. 27309487

[77] Cuzon G. , Naas T. , and Nordmann P. , Functional Characterization of Tn4401, a Tn3-Based Transposon Involved inblaKPCGene Mobilization, Antimicrobial Agents and Chemotherapy. (2011) 55, no. 11, 5370–5373, 10.1128/aac.05202-11, 21844325. PMC3195030 21844325

[78] Muteeb G. , Kazi R. N. A. , Aatif M. , Azhar A. , El Oirdi M. , and Farhan M. , Antimicrobial Resistance: Linking Molecular Mechanisms to Public Health Impact, SLAS Discovery. (2025) 33, 100232, 10.1016/j.slasd.2025.100232. 40216324

[79] Ciric L. , Jasni A. , de Vries L. E. , Agersø Y. , Mullany P. , and Roberts A. P. , The Tn916/Tn1545 Family of Conjugative Transposons, Bacterial Integrative Mobile Genetic Elements, 2022, CRC Press, 153–170, https://discovery.ucl.ac.uk/id/eprint/1383521/3/1383521_Azmiza%252520Jasni_Book%252520Chapter.pdf.

[80] Deng Y. , Bao X. , Ji L. , Chen L. , Liu J. , Miao J. , Chen D. , Bian H. , Li Y. , and Yu G. , Resistance integrons: Class 1, 2 and 3 Integrons, Annals of Clinical Microbiology and Antimicrobials. (2015) 14, no. 1, 10.1186/s12941-015-0100-6, 26487554. PMC4618277 26487554

[81] Lahiri D. , Nag M. , Dutta B. , Dey A. , and Ray R. R. , Bacterial Extracellular Polysaccharides in Biofilm Formation and Function, Application of Biofilms in Applied Microbiology, 2022, Elsevier, 1–23, 10.1016/B978-0-323-90513-8.00003-0.

[82] Abu-Shoura E. J. I. , Hudu S. A. , and AL-Quadan T. F. , Exploring Genetic and Phenotypic Factors Contributing to Urethral Catheter Biofilm Formation in Hospitalised Patients in Jordan, Biomedical and Pharmacology Journal. (2024) 17, no. 2, 1125–1134, 10.13005/bpj/2928.

[83] Almatroudi A. , Biofilm resilience: Molecular Mechanisms Driving Antibiotic Resistance in Clinical Contexts, Biology. (2025) 14, no. 2, 10.3390/biology14020165, 40001933. PMC11852148 40001933

[84] Lee Y. J. and Lee Y. J. , Characterization of Biofilm Producing Coagulase-Negative Staphylococci Isolated From Bulk Tank Milk, Veterinary Sciences. (2022) 9, no. 8, 10.3390/vetsci9080430, 36006345. PMC9416249 36006345

[85] Araújo D. , Silva A. R. , Fernandes R. , Serra P. , Barros M. M. , Campos A. M. , Oliveira R. , Silva S. , Almeida C. , and Castro J. , Emerging Approaches for Mitigating Biofilm-Formation-Associated Infections in Farm, Wild, and Companion Animals, Pathogens. (2024) 13, no. 4, 10.3390/pathogens13040320, 38668275. PMC11054384 38668275

[86] Iaconis A. , De Plano L. M. , Caccamo A. , Franco D. , and Conoci S. , Anti-Biofilm Strategies: A Focused Review on Innovative Approaches, Microorganisms. (2024) 12, no. 4, 10.3390/microorganisms12040639, 38674584. PMC11052202 38674584

[87] Roy R. , Tiwari M. , Donelli G. , and Tiwari V. , Strategies for Combating Bacterial Biofilms: A Focus on Anti-Biofilm Agents and Their Mechanisms of Action, Virulence. (2018) 9, no. 1, 522–554, 10.1080/21505594.2017.1313372, 28362216. PMC5955472 28362216

[88] Hawas S. , Verderosa A. D. , and Totsika M. , Combination Therapies for Biofilm Inhibition and Eradication: A Comparative Review of Laboratory and Preclinical Studies, Frontiers in Cellular and Infection Microbiology. (2022) 12, 850030, 10.3389/fcimb.2022.850030, 35281447. PMC8915430 35281447

[89] Makhlouf Z. , Ali A. A. , and Al-Sayah M. H. , Liposomes-Based Drug Delivery Systems of Anti-Biofilm Agents to Combat Bacterial Biofilm Formation, Antibiotics. (2023) 12, no. 5, 10.3390/antibiotics12050875, 37237778. PMC10215698 37237778

[90] Sharma D. , Singh I. , Sharma J. , Verma I. K. , and Ratn A. , Bacteriophage Therapy to Combat Microbial Infections and Antimicrobial Resistance, Journal of Basic Microbiology. (2025) 65, no. 11, e70090, 10.1002/jobm.70090. 40757460

[91] Guo Z. , Liu M. , and Zhang D. , Potential of Phage Depolymerase for the Treatment of Bacterial Biofilms, Virulence. (2023) 14, no. 1, 2273567, 10.1080/21505594.2023.2273567, 37872768. PMC10621286 37872768

[92] Topka-Bielecka G. , Dydecka A. , Necel A. , Bloch S. , Nejman-Faleńczyk B. , Węgrzyn G. , and Węgrzyn A. , Bacteriophage-Derived Depolymerases Against Bacterial Biofilm, Antibiotics. (2021) 10, no. 2, 10.3390/antibiotics10020175, 33578658. PMC7916357 33578658

[93] Abedon S. T. , Phage Therapy: Combating Evolution of Bacterial Resistance to Phages, Viruses. (2025) 17, no. 8, 10.3390/v17081094, 40872808. PMC12390681 40872808

[94] Selim H. M. R. M. , Gomaa F. A. M. , Alshahrani M. Y. , Morgan R. N. , and Aboshanab K. M. , Phage Therapeutic Delivery Methods and Clinical Trials for Combating Clinically Relevant Pathogens, Therapeutic Delivery. (2025) 16, no. 3, 247–269, 10.1080/20415990.2024.2426824, 39545771. PMC11875505 39545771

[95] Al-Madboly L. A. , Aboulmagd A. , El-Salam M. A. , Kushkevych I. , and El-Morsi R. M. , Microbial Enzymes as Powerful Natural Anti-Biofilm Candidates, Microbial Cell Factories. (2024) 23, no. 1, 10.1186/s12934-024-02610-y, 39710670. PMC11664836 39710670

[96] Acquaviva R. , D’Angeli F. , Malfa G. A. , Ronsisvalle S. , Garozzo A. , Stivala A. , Ragusa S. , Nicolosi D. , Salmeri M. , and Genovese C. , Antibacterial and Anti-Biofilm Activities of Walnut Pellicle Extract (Juglans regia L.) Against Coagulase-Negative Staphylococci, Natural Product Research. (2021) 35, no. 12, 2076–2081, 10.1080/14786419.2019.1650352, 31397177. 31397177

[97] Chusri S. , Tongrod S. , Saising J. , Mordmuang A. , Limsuwan S. , Sanpinit S. , and Voravuthikunchai S. P. , Antibacterial and Anti-Biofilm Effects of a Polyherbal Formula and Its Constituents Against Coagulase-Negative and -positive Staphylococci Isolated From Bovine Mastitis, Journal of Applied Animal Research. (2017) 45, no. 1, 364–372, 10.1080/09712119.2016.1193021.

[98] Piekarska-Radzik L. , Milala J. , Klewicki R. , Sójka M. , Żyżelewicz D. , Matysiak B. , and Klewicka E. , Extract from Rosa spp. As a Factor Influencing the Growth Rate of Coagulase-Negative Staphylococcus Strains, Molecules. (2025) 30, no. 7, 10.3390/molecules30071443, 40286034. PMC11990744 40286034

[99] Chatzigeorgiou K.-S. , Siafakas N. , Petinaki E. , Argyropoulou A. , Tarpatzi A. , Bobola M. , Paniara O. , Velegraki A. , and Zerva L. , Identification of Staphylococci by Phoenix: Validation of a New Protocol and Comparison With Vitek 2, Diagnostic Microbiology and Infectious Disease. (2010) 68, no. 4, 375–381, 10.1016/j.diagmicrobio.2010.08.010, 21094424. 21094424

[100] Mishra A. , Tabassum N. , Aggarwal A. , Kim Y.-M. , and Khan F. , Artificial Intelligence-Driven Analysis of Antimicrobial-Resistant and Biofilm-Forming Pathogens on Biotic and Abiotic Surfaces, Antibiotics. (2024) 13, no. 8, 10.3390/antibiotics13080788, 39200087. PMC11351874 39200087

[101] Uelze L. , Grützke J. , Borowiak M. , Hammerl J. A. , Juraschek K. , Deneke C. , Tausch S. H. , and Malorny B. , Typing Methods Based on Whole-Genome Sequencing Data, One Health Outlook. (2020) 2, no. 1, 10.1186/s42522-020-0010-1, 33829127. PMC7993478 33829127

[102] Zhang X. , Zhou Y. , Fu L. , Zhou L. , Cheng X. , Zhang W. , and Tan Z. , WGS Analysis of *Staphylococcus warneri* Outbreak in a Neonatal Intensive Care Unit, Infection and Drug Resistance. (2024) 17, 4279–4289, 10.2147/IDR.S473525, 39377031. PMC11457789 39377031

[103] Hajhamed N. M. , Mohamed N. S. , Abdalla A. E. , Ebrahim R. , Mohammed S. I. , Bakheit A. M. , Azhary A. , Ahmed A. E. , Abdelbagi A. , Ali M. S. E. , Omer R. A. , Siddig E. E. , Ahmed A. , Hamida M. E. , and Salim M. A. , Current Status and Recent Trends in Innovative Tactics and the One Health Approach to Address the Challenge of Methicillin-Resistant *Staphylococcus aureus* Infections: A Comprehensive Review, Discover Medicine. (2025) 2, no. 1, 1–22, 10.1007/s44337-025-00300-1.

[104] Paramitadevi Y. V. , Priadi C. R. , Rahmatika I. , Rukmana A. , and Moersidik S. S. , Integration of Water, Sanitation, and Hygiene Program With Biosecurity: A One Health Approach to Reduce the Prevalence and Exposure of Antibiotic-Resistant Bacteria in the Livestock Community, International Journal of One Health. (2023) 9, 181–193, 10.14202/IJOH.2023.181-193.

[105] Lappan R. , Chown S. L. , French M. , Perlaza-Jiménez L. , Macesic N. , Davis M. , Brown R. , Cheng A. , Clasen T. , and Conlan L. , Towards Integrated Cross-Sectoral Surveillance of Pathogens and Antimicrobial Resistance: Needs, Approaches, and Considerations for Linking Surveillance to Action, Environment International. (2024) 192, 109046, 10.1016/j.envint.2024.109046. 39378692

